# Supplementary material for: Antiobesity and Hypolipidemic Potential of Nitraria retusa Extract in Overweight/Obese Women: A Randomized, Double-Blind, Placebo-Controlled Pilot Study
Source: Nutrients. 2024 Jan 21;16(2):317. doi: 10.3390/nu16020317 (PMC10818277; doi:10.3390/nu16020317)
Supplement: Supplementary file 1 [file nutrients-16-00317-s001.zip › nutrients-2760766-supplementary.pdf]

nutrients

Supplementary Data

Anti-obesity and Hypolipidemic Potential of *Nitraria retusa* Extract in overweight/obese Women: A Randomized, Double- Blind, placebo- Controlled Pilot Study

Aicha Laouani [1,2], Hana Nasrallah [1,2], Awatef Sassi [1,2], Farhana Ferdousi [3,4], Feten Zar Kalai [4,5], Yosra Hasni [6], Hiroko Isoda [3,4,7,*] and Saad Saguem [1,2,*]

1 Laboratory of Metabolic Biophysics and Applied Pharmacology, Faculty of Medicine, University of Sousse, Tunisia; laouani_aicha@yahoo.fr (A.L.); hananasrallah.hn@gmail.com (H.N.); awatefsassi@yahoo.fr (A.S.); khaled_saguem@yahoo.fr (S.S.)
2 USCR Analytical Platform UHPLC-MS &Research in Medicine and Biology, Faculty of Medicine, University of Sousse, Tunisia
3 Faculty of Life and Environmental Sciences, University of Tsukuba, Tsukuba 305-8572, Japan; ferdousi.farhana.fn@u.tsukuba.ac.jp (F.F.); isoda.hiroko.ga@u.tsukuba.ac.jp (H.I.)
4 Alliance for Research on the Mediterranean and North Africa (ARENA), University of Tsukuba, Tsukuba 305-8572, Japan
5 Laboratory of Aromatic and Medicinal Plants, Center of Biotechnology, Technopark of Borj Cedria, BP 901, 2050 Hammam-Lif, Tunisia; zarfeten@gmail.com (F.Z.K.)
6 Endocrinology-Diabetology Department, Farhat Hached Hospital, Sousse, Tunisia; yosrahasnielabed@gmail.com (Y.H.)
7 Open Innovation Laboratory for Food and Medicinal Resource Engineering (FoodMed-OIL), National Institute of Advanced Industrial Science and Technology (AIST), Tsukuba 305-8577, Japan
* Correspondence: isoda.hiroko.ga@u.tsukuba.ac.jp (H.I.); khaled_saguem@yahoo.fr (S.S.)

Supplementary Table S1: Means of change values ± SD per parameter before and after 12 week NRE and placebo intake: Body weight, Body composition, Anthropometric parameters and lipid profile of overweight/ obese participants with BMI< 29.9 Kg/m^2.

	**Δ NRE (N=8)**	**Δ Placebo (N=3)**	***p* value[a]**
**Weight**	-1.01 ±0.67	+0.83 ±1.19	0.048
**BMI**	-0.35 ±0.18	-0.06 ±0.75	0.44
**WC**	-3.43 ±0.68	-1.33 ±4.95	0.609
**BF**	-0.46 ±0.35	+0.43 ±0.34	0.279
**BM**	+0.15 ±0.108	-0.13 ±0.24	0.246
**BM/BF**	+0.015 ±0.01	-0.227 ±0.029	0.197
**BW**	+0.36 ±0.25	-0.4 ±0.36	0.166
**WC/HC**	-0.25 ±0.006	+0.044 ±0.04	0.22
**MTC**	-2.41 ±0.95	+1.83 ±1.16	0.01
**MAC**	-1±0.53	+1.16 ±2.52	0.69
**TG, mmol/L**	-0.0217 ±0.086	+0.06 ±0.04	0.41
**HDL, mmol/L**	-0.038 ±0.06	- 0.026 ±0.075	0.75
**LDL, mmol/L**	-0.43 ±0.22	+0.136 ±0.173	0.09
**TC, mmol/L**	-0.32 ±0.25	+0.126 ±0.18	0.286

[a]Between-group differences (NRE vs. Placebo) in the parameters were assessed by independent samples t-test (parametric and equal variances), Welch's t-test (unequal variances) and Mann-Whitney U test (nonparametric)

Supplementary Table S2: Means of change values ± SD per parameter before and after 12 week NRE and placebo intake: Body weight, Body composition, Anthropometric parameters and lipid profile of overweight/ obese participants with BMI ≥ 30 kg/m².

	**Δ NRE (N=22)**	**Δ Placebo (N=10)**	***p* value[a]**
**Weight**	-2.86 ±0.61	-0.18 ±0.43	<0.001
**BMI**	-1.03 ±0.22	-0.088 ±0.18	0.01
**WC**	-2.87 ±1.25	-0.66 ±0.63	<0.001
**BF**	-1.26 ±0.29	-0.105 ±0.28	0.09
**BM**	+0.37±0.2	+0.2±0.22	0.11
**BM/BF**	0.028 ±0.009	0.0058 ±0.008	0.07
**BW**	1.1 ±0.2	0.2 ±0.204	0.006
**WC/HC**	0.0054 ±0.0039	-0.006 ±0.004	0.34
**MTC**	-3.37 ±0.94	-1.05 ±0.78	0.039
**MAC**	-1.81±0.41	-1.22 ±0.66	0.37
**TG, mmol/L**	-0.038±0.14	+0.1 ±0.0088	0.016
**HDL, mmol/L**	+0.03 ±0.031	+0.065 ±0.06	0.78
**LDL, mmol/L**	0.38 ±0.23	0.027 ±0.225	0.514
**TC, mmol/L**	0.223 ±0.18	0.14 ±0.29	0.93

[a]Between-group differences (NRE vs. Placebo) in the parameters were assessed by independent samples t-test (parametric and equal variances), Welch's t-test (unequal variances) and Mann-Whitney U test (nonparametric)

Supplementary Table S3: Means of change values ± SD per parameter before and after 12 week NRE and placebo intake: Body weight, Body composition, Anthropometric parameters and lipid profile of overweight/ obese participants with age < 40 years.

Parametere	Δ<br>**NRE (17)**	Δ<br>**Placebo (4)**	*p* **value**[a]
**Weight**	-2.51 ±0.71	+1.05 ±0.79	0.003
**BMI**	-0.9 ±0.27	0.1 ±0.54	0.088
**WC**	-3.6 ±0.89	-1.25 ±3.59	0.333
**BF**	-1.17 ±0.33	0.11 ±0.43	0.034
**BM**	+0.42 ±0.15	-0.125 ±0.17	0.056
**BM/BF**	+0.0311 ±0.009	-0.148 ±0.24	0.061
**BW**	+1 ±0.27	-0.1 ±0.43	0.006
**WC/HC**	-0.115 ±0.006	0.29 ±0.33	0.019
**MTC**	-2.87 ±1.17	+0,5 ±1.63	0.129
**MAC**	-1.43 ±0.51	+0.25 ±2	0.36
**TG, mmol/L**	-0.067 ±0.07	+0.03 ±0.086	0.21
**HDL, mmol/L**	+0.08 ±0.27	+0.1 ±0.75	0.8
**LDL, mmol/L**	-0.29 ±0.126	+0.19 ±0.29	0.46
**TC, mmol/L**	+0.41±0.1	+0.31 ±0.31	0.31

[a]Between-group differences (NRE vs. Placebo) in the parameters were assessed by independent samples t-test (parametric and equal variances), Welch's t-test (unequal variances) and Mann-Whitney U test (nonparametric)

Supplementary Table S4: Means of change values ± SD per parameter before and after 12 week NRE and placebo intake: Body weight, Body composition, Anthropometric parameters and lipid profile of overweight/ obese participants with age> 40 years.

	Δ NRE (N=13)	Δ Placebo (N=9)	p value[a]
**Weight**	-1.48 ±0.39	-0.42 ±0.45	0.048
**BMI**	-0.53 ±0.17	-0.17 ±0.187	0.044
**WC**	-2.43 ±1.34	-0.62 ±0.617	0.037
**BF**	-0.58 ±0.32	-0.01 ±0.29	0.139
**BM**	0.083±0.19	0.23±0.24	0.512
**BM/BF**	0.012 ±0.008	0.0036 ±0.008	0.49
**BW**	0.5 ±0.18	0.125 ±0.199	0.203
**WC/HC**	-0.0028 ±0.015	-0.0049 ±0.004	0.301
**MTC**	-3.08 ±0.59	-0.5 ±0.829	0.01
**MAC**	-0.5±0.61	-1.06 ±0.73	0.36
**TG, mmol/L**	-0.075 ±0.11	0.125 ±0.09	0.009
**HDL, mmol/L**	-0.026 ±0.06	0.033 ±0.06	0.816
**LDL, mmol/L**	-0.15 ±0.36	-0.013 ±0.22	0.809
**TC, mmol/L**	-0.18 ±0.36	0.05 ±0.296	0.934

[a]Between-group differences (NRE vs. Placebo) in the parameters were assessed by independent samples t-test (parametric and equal variances), Welch's t-test (unequal variances) and Mann-Whitney U test (nonparametric)

Supplementary Table S5: Means of change values ± SD per parameter before and after 12 week NRE and placebo intake: Body weight, Body composition, Anthropometric parameters and lipid profile of overweight/ obese participants participants with BMI < 30 and age < 40.

	Δ NRE (N=4)	Δ Placebo (N=2)	p value[a]
**Weight**	-1.13 ±0.6	+1.3 ±1.9	0.128
**BMI**	-0.36 ±0.34	-0.1 ±1.3	0.74
**WC**	-2.83 ±1.3	-2 ±8.5	0.81
**BF**	-0.73 ±0.67	0.67 ±0.41	0.092
**BM**	0.1 ±0.208	-0.2 ±0.4	0.59
**BM/BF**	0.02 ±0.022	-0.042 ±0.04	0.244
**BW**	0.56 ±0.49	-0.65 ±0.45	013
**WC/HC**	-0.022 ±0.01	0.07 ±0.056	0.315
**MTC**	-2 ±2.08	2 ±2	0.201
**MAC**	-0.16 ±0.16	1.75 ±4.25	0.36
**TG, mmol/L**	-0.0025 ±0.033	0.09 ±0.07	0.256
**HDL, mmol/L**	-0.0067 ±0.05	0.09 ±0.07	0.921
**LDL, mmol/L**	-0.11±0.2	0.105 ±0.29	0.577
**TC, mmol/L**	0.14 ±0.147	0.24 ±0.26	0.751

[a]Between-group differences (NRE vs. Placebo) in the parameters were assessed by independent samples t-test (parametric and equal variances), Welch's t-test (unequal variances) and Mann-Whitney U test (nonparametric)

Supplementary Table S6: Means of change values ± SD per parameter before and after 12 week NRE and placebo intake: Body weight, Body composition, Anthropometric parameters and lipid profile of overweight/ obese participants with BMI ≥30.0 Kg/ m^{2}and age<40 years.

	Δ<br>NRE (N=13)	Δ<br>Placebo (N=2)	p value[a]
**Weight**	-3.34 ±0.93	0.8 ±0.1	<0.001
**BMI**	-1.22 ±0.32	0.3 ±0.1	0.103
**WC**	-4.1 ±1.24	-0.5 ±2	0.041
**BF**	-1.44 ±0.35	-0.45 ±0.35	0.17
**BM**	0.62 ±0.17	-0.05 ±0.05	0.07
**BM/BF**	0.037 ±0.009	0.0132 ±0.014	0.214
**BW**	1.26 ±0.31	+0.45 ±0.55	0.33
**WC/HC**	-0.005 ±0.007	-0.011 ±0.008	0.613
**MTC**	-3.4 ±1.41	-2 ±2	0.705
**MAC**	-2.2 ±0.58	-1.25 ±1.25	0.842
**TG, mmol/L**	0.02 ±0.21	-0.03 ±0.18	0.826
**HDL, mmol/L**	0.038 ±0.033	+0.11 ±0.17	0.909
**LDL, mmol/L**	0.33 ±0.28	+0.28 ±0.64	0.54
**TC, mmol/L**	0.09 ±0.139	0.38 ±0.73	0.377

[a]Between-group differences (NRE vs. Placebo) in the parameters were assessed by independent samples t-test (parametric and equal variances), Welch's t-test (unequal variances) and Mann-Whitney U test (nonparametric)

Supplementary Table S7: Means of change values ± SD per parameter before and after 12 week NRE and placebo intake: Body weight, Body composition, Anthropometric parameters and lipid profile of overweight/ obese participants with BMI≥ 30.0 and age>40.

	Δ NRE (N=9)	Δ Placebo (N=8)	p value[a]
**Weight**	-2.06 ±0.29	-0.47 ±0.51	0.005
**BMI**	-0.73 ±0.23	-0.2 ±0.214	0.034
**WC**	-0.83 ±2.48	-0.71 ±0.7	0.159
**BF**	-0.96 ±0.54	-0.0006 ±0.33	0.254
**BM**	-0.033 ±0.39	0.27 ±0.28	0.9
**BM/BF**	0.01 ±0.01	0.0037 ±0.009	0.588
**BW**	0.83 ±0.06	0.128 ±0.23	0.222
**WC/HC**	0.022 ±0.02	-0.0043 ±0.0057	0.87
**MTC**	-3.33 ±1.3	-0.78 ±0.89	0.035
**MAC**	-1.16 ±0.33	-1.21 ±0.82	0.209
**TG, mmol/L**	-0.136 ±0.164	0.14 ±0.105	0.006
**HDL, mmol/L**	0.0167 ±0.07	0.052 ±0.074	0.72
**LDL, mmol/L**	0.48 ±0.47	-0.0443 ±0.25	0.18
**TC, mmol/L**	0.43 ±0.46	0.071 ±0.34	0.48

[a]Between-group differences (NRE vs. Placebo) in the parameters were assessed by independent samples t-test (parametric and equal variances), Welch's t-test (unequal variances) and Mann-Whitney U test (nonparametric)
